# Apoferritin Amyloid-Fibril Directed the In Situ Assembly and/or Synthesis of Optical and Magnetic Nanoparticles

**DOI:** 10.3390/nano11010146

**Published:** 2021-01-08

**Authors:** Rocío Jurado, Natividad Gálvez

**Affiliations:** Department of Inorganic Chemistry, University of Granada, 18071 Granada, Spain; rociojp@ugr.es

**Keywords:** amyloid fibrils, apoferritin protein, β-lactoglobulin, magnetic nanoparticles, gold nanoparticles, MRI and optical imaging, bionanomaterials

## Abstract

The coupling of proteins that can assemble, recognise or mineralise specific inorganic species is a promising strategy for the synthesis of nanoscale materials with a controllable morphology and functionality. Herein, we report that apoferritin protein amyloid fibrils (APO) have the ability to assemble and/or synthesise various metal and metal compound nanoparticles (NPs). As such, we prepared metal NP–protein hybrid bioconjugates with improved optical and magnetic properties by coupling diverse gold (AuNPs) and magnetic iron oxide nanoparticles (MNPs) to apoferritin amyloid fibrils and compared them to the well-known β-lactoglobulin (BLG) protein. In a second approach, we used of solvent-exposed metal-binding residues in APO amyloid fibrils as nanoreactors for the in situ synthesis of gold, silver (AgNPs) and palladium nanoparticles (PdNPs). Our results demonstrate, the versatile nature of the APO biotemplate and its high potential for preparing functional hybrid bionanomaterials. Specifically, the use of apoferritin fibrils as vectors to integrate magnetic MNPs or AuNPs is a promising synthetic strategy for the preparation of specific contrast agents for early in vivo detection using various bioimaging techniques.

## 1. Introduction

Great efforts are currently underway to develop new probes for noninvasive imaging techniques such as high-resolution magnetic resonance imaging (MRI) or optical imaging [1,2,3,4]. While there have been great advances, biomedical imaging still suffers from problems associated with resolution, sensitivity, speed, and penetration depth [5,6]. Inorganic NPs are widely considered to have potential as structural and functional building-blocks for new biomedical imaging applications [2]. Due to the unique optical properties of AuNPs, for example, surface plasmon resonance (SPR), they can readily be used to enhance optical imaging techniques [6]. Recent innovations in optical imaging, including dark field, bright field, DIC microscopy, photothermal and photoluminescence detection methods, among others, can be used to view and to track the intracellular locations and behaviour of AuNPs [6]. The anisotropy of certain morphologies, such as nanowires, nanoprisms or nanorods, generates two SPR absorption bands corresponding to the transverse and longitudinal axes, the latter usually at low energies, in the infrared region [7,8,9]. AuNPs properties, including plasmonic properties, targeting, and bio-compatibility, have turned them into incredibly useful nanomaterials with a wide range of chemical and biological applications [2,6,10,11,12,13,14]. Recently, a significant amount of effort has focused on assembling NPs into sophisticated structures amenable to practical uses, especially in bio-imaging [2,6,12]. One example of molecular templating strategies includes the use of amyloid fibrils [15,16].

Furthermore, iron oxide-based MNPs have been proposed as specific contrast agents for MRI to improve detection at early stages of a disease, and to diminish the acquisition times of in vivo imaging [3,4,17]. The hypointense effect exhibited by these particles in T2 and T2*-weighted imaging sequences produces a greater contrast in MR images. Several properties of these inorganic nanomaterials are thought to depend not only on their size, shape and chemical composition but also to a large extent on the spatial arrangement of these building blocks relative to each other within a material [3,18,19]. An early study by Duan et al. [20] suggested that hydrophilic surface coatings, developed for in vivo applications, contribute greatly to the resulting MRI contrast effect. Accordingly, biocompatible and hydrophilic protein scaffolds facilitate water’s access to the magnetic core [21]. The influence of particle size on relaxation rates has been widely studied as another key factor to help improve contrast agent performance [17,22]. It has been shown the saturation magnetisation (M_S_) and MRI signal to increase with nanoparticle size [23]. One means of improving magnetic properties to enhance the MRI contrast effect is by assembling NPs into sophisticated structures, for example, magnetic clusters comprised of small MNPs, thus increasing the effective magnetic size [19]. Along the same lines, protein fibrils can be used as templates to achieve a high degree of spatial alignment of the NPs, thereby increasing the magnetic anisotropy and, consequently, the magnetic properties of the resulting nanostructure [24,25].

The ferritin protein has an essential metabolic role involving iron storage and homeostasis, in practically all life forms [26,27]. The genuine APO, the iron-free ferritin molecule, is a hollow nanocage protein composed of 24 polypeptide subunits (Mn ∼480 kDa) [28]. Bioengineered ferritin nanocages have been used as nanoprobes for MRI [29] or the bimodal imaging of tumours with high sensitivity and specificity in live mice using SPECT and MRI a single intravenous injection [30]. Similarly, maghemite NPs encapsulated inside ferritin nanocages exhibited useful properties to act as in vivo long-term MRI contrast agents [31,32].

Amyloids are insoluble aggregates formed in vivo from misfolded proteins [33] and are gaining relevance as biomaterial templates with potential in a number of applications, such as rapid diagnostic tools [34,35], cell culture substrates [36], drug delivery systems [37], or therapeutics [38], thanks to their combination of physical properties with biological compatibility [39]. For example, the AuNPs–β-lactoglobulin amyloid fibril bioconjugate, was shown to enhance the transport of iron NPs in living cells [40].

In previous work, we described the formation of APO amyloid fibrils and their potential for use as new templates in the design of chiral functional nanomaterials [41,42]. In the present study, we have used APO and BLG fibril proteins as biomaterial templates to integrate AuNPs (with different morphologies) and MNPs to form metal NPs–protein hybrid bioconjugates with improved physical properties. We used BLG fibril protein as a valuable control model protein. In a second approach, we used solvent-exposed metal-binding residues in APO amyloid fibrils as nanoreactors for the in situ synthesis of AuNPs, AgNPs and PdNPs bioconjugates. Amyloid protein structures not only have a high affinity for a wide range of metals, but can also incorporate of a high number of NPs per protein [40,43]. Finally, a final advantage of protein fibrils is that have homogeneous, reproducible and easily tuned diameters. All of this, coupled with the need to develop synthetic methods of reliably producing new contrast agents, means that APO fibrils represent a promising strategy for the preparation of specific contrast agents for diverse bio-optical imaging techniques.

## 2. Materials and Methods

### 2.1. Preparation of APO and BLG Amyloid Fibrils

Horse spleen apoferritin and BioPURE bovine β-lactoglobulin proteins were purchased from MERCK LIFE SCIENCE SLU (Madrid, Spain). Purified protein solutions (0.1 wt %) were adjusted to pH 2 (0.1 M HCl) before heat treatment (90 °C, in hermetically sealed glass tubes) over an incubation time period of 9 h. After heat treatment, the glass tubes were cooled in an ice bath to quench the aggregation process.

### 2.2. Metal Nanoparticle Synthesis

#### 2.2.1. Gold Nanosphere (AuNSs) Synthesis

Chloroauric acid was purchased from Sigma Aldrich and used without any further purification. Five milliliters of a 1.0 mM aqueous solution of HAuCl_4_ was stirred and heated to boiling on a hot plate. Once it began to boil, 500 μL of a 38.8 mM aqueous solution of Na_3_C_6_H_5_O_7_ was added. The mixture was boiled and stirred continuously for about 10 min until it produced a deep red colour [44]. It was then allowed to cool to room temperature. The AuNSs were characterized by UV-vis spectroscopy (Varian Cary-100 spectrophotometer, Agilent Technologies Spain SL, Madrid, Spain) and transmission electron microscopy (TEM) with a LIBRA 120 PLUS operating at 120 keV.

#### 2.2.2. Gold Nanorods (AuNRs) Synthesis

We employed a seed-mediated growth method to prepare AuNRs [45]. The gold seed was prepared as described elsewhere. A 10 mM solution of NaBH_4_ was freshly prepared. We added 600 µL of the NaBH_4_ solution to a HAuCl_4_ solution (1 mL, 1 mM) in 0.2 M cetyltrimethylammonium bromide (CTAB) under rapid stirring. When seeds were formed, the solution changed from yellow to light brown. For the NRs synthesis, a AgNO_3_ solution (100 µL, 8 mM) was added to an HAuCl_4_ solution (5 mL,1 mM) in 0.2 M CTAB, followed by the addition of ascorbic acid solution (70 µL, 78.8 mM), and the resulting mixture was stirred until it became transparent. Next, we added 24 µL of the seed solution, and the growth solution was mixed thoroughly at 30 °C for 7 days. The resulting NRs were purified by centrifugation at 13,000 rpm and then redispersed in water. The NRs were finally characterised by UV-vis spectroscopy and transmission electron microscopy (TEM).

#### 2.2.3. Maghemite Magnetic Nanoparticles (MNPs) Synthesis

Magnetite NPs were prepared by co-precipitating iron salts in solution following a slightly modified Massart’s method [24]. Compared to nonpolar organometallic routes, aqueous synthesis is more reproducible, cheaper, nontoxic and the products have a high aqueous stability and biological compatibility. The oxidation of magnetite in acidic conditions produces colloidal of maghemite (γ-Fe_2_O_3_) NPs, which are chemically stable at pH 2.

Briefly, magnetite NPs were prepared by the co-precipitation of Fe^2+^ [(NH_4_)_2_Fe(SO_4_)_2_] and Fe^3+^ [Fe(NO_3_)_3_] salts at a stoichiometry of 0.5 in an alkaline medium. All solutions were carefully deaerated with argon before reaction. After the oxidation of magnetite to maghemite with 1 M HClO_4_, we obtained a sol (stable colloid solution) of maghemite NPs measuring 8 ± 2 nm at pH 2 with an iron concentration of 0.1 M.

### 2.3. APO and BLG Amyloid Fibril-Biotemplated AuNPs and MNPs

To prepare the AuNSs and AuNRs bioconjugates, we added 500 µL AuNSs or AuNRs solution was added to 1 mL of APO or BLG fibril solution at pH 8 and incubated for 24 h. The MNP bioconjugate was prepared by adding an acidic colloid of 8 nm maghemite NPs (200 μL, 1 mM) to 1 mL of APO or BLG amyloid fibril solution at pH 8 and incubating for 24 h. The maghemite NPs were sonicated prior to mixing to prevent particle aggregation.

### 2.4. Magnetic Measurements

Magnetic measurements were performed on lyophilised samples using a magnetometer (Quantum Design MPMS-XL-5, Quantum Design Europe, Darmstadt Germany) equipped with a SQUID sensor.

### 2.5. Metal-Binding APO Fibrils as Nanoreactors for the Preparation of Metal NPs–Protein Bioconjugates

AuNPs–, AgNP– and PdNP–protein bioconjugates were prepared by initial mixing of a 0.1 wt % solution of the APO protein fibrils (500 μL) with 0.004 M solutions of HAuCl_4_, AgNO_3_, and PdCl_2_ salts, respectively. After 15 min of incubation at room temperature (23 °C), 6 μL of reducing agent (NaBH_4_ 0.01M) was added and the solution incubated for 1 h.

### 2.6. TEM Samples

Samples were prepared by placing a drop onto a carbon-coated Cu grid. Electron micrographs were taken with a LIBRA 120 PLUS microscope operating at 120 keV. High-resolution scanning transmission electron microscopy (HR-STEM), high-angle annular dark-field scanning transmission electron microscopy (HAADF-STEM) and energy-dispersive X-ray spectroscopy (EDXS) maps were obtained with a FEI TITAN G2 microscope.

## 3. Results and Discussion

**Scheme 1:**
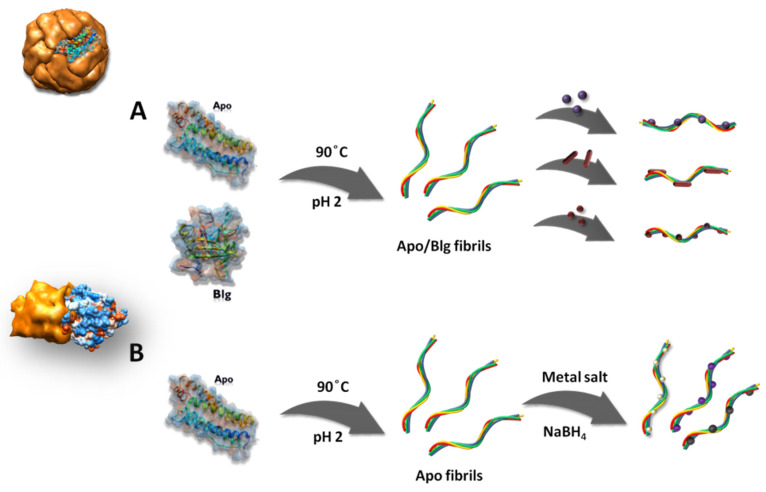
Schematic illustration of metal NPs–protein bioconjugate formation. (**A**) Strategy 1: apoferritin protein amyloid fibrils (APO) and β-lactoglobulin (BLG) protein fibrils assemble different metal nanoparticles (NPs): AuNSs, AuNRs and magnetic iron oxide nanoparticles (MNPs). (**B**) Strategy 2: metal-binding APO fibril precursors are used for the in situ synthesis of NPs.

Scheme q illustrates the two synthetic strategies used to prepare the metal NP–protein bioconjugates. In the first strategy, APO and BLG protein fibril templates were prepared previously through a temperature-induced self-assembly process [41]. AuNSs, AuNRs and MNPs were synthesised according to methods in the literature (see Materials and Mehtods) and were assembled using APO and BLG amyloid fibrils (Scheme 1A). In a second approach, metal-binding fibril precursors were used as nanoreactors for the synthesis of protein bioconjugates (Scheme 1B), via the in situ reduction of a metal salt.

### 3.1. APO and BLG Amyloid Fibril-Biotemplated AuNPs

**Figure 1:**
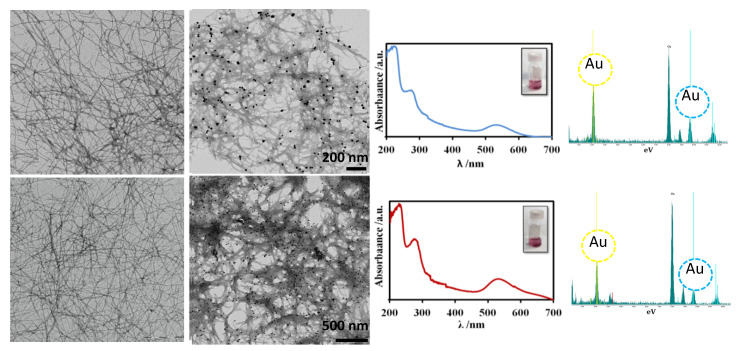
From left to right: transmission electron microscopy (TEM)-contrasted images, UV-vis spectra and the corresponding EDXS elemental analysis for the AuNSs–APO (**top row**) and the AuNSs–BLG bioconjugates (**bottom row**). TEM images further to the left are the pristine APO–amyloid fibrils (**top row**) and BLG–amyloid fibrils (**bottom row**).

AuNSs–protein bioconjugates were prepared after incubating of AuNSs (average particle size distribution of 12 ± 2 nm, Figure S1) with protein fibrils for 24 h. Figure 1 shows negatively stained TEM images of the pristine APO– and BLG–amyloid fibrils, and the AuNS–APO and AuNS–BLG protein bioconjugates. The figure also shows semiflexible fibrils with an average diameter of 7 nm and a contour length distribution ranging from one to several microns are shown, in accordance with earlier work [31]. The incorporation of the AuNSs onto the fibrils was clearly successful because of no free NPs were observed in the medium for either proteins. Furthermore, the presence of the fibril templates prevented, for long periods, the irreversible aggregation of the metal NPs and their subsequent precipitation (Figure 1), in contrast to the metal NPs precipitation in the protein fibril-free medium after 1 h.

The UV-visible absorption spectra recorded for AuNSs–APO and AuNSs–BLG fibrils displayed the typical SPR absorption band for 12 nm isolated spherical gold NPs centered at 530 nm (Figure 1). Both sample solutions were characterised by a transparent pinkish/purple colour (Figure 1, inset). EDXS analysis confirmed the presence of gold (Figure 1).

**Figure 2:**
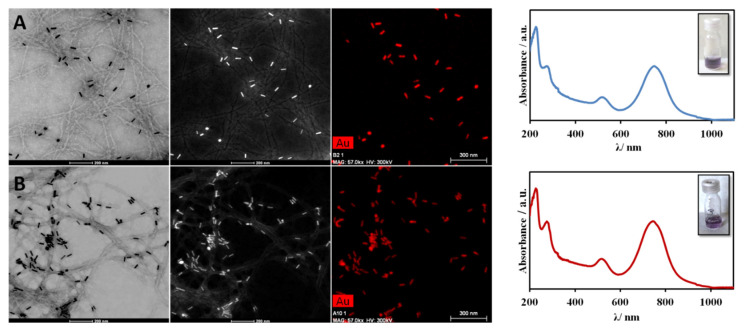
From left to right: Bright-field image, high-angle annular dark-field scanning transmission electron microscopy (HAADF-STEM) image, the corresponding EDX elemental mapping of gold and the UV-vis spectra of the AuNR–APO (top row, (**A**)) and AuNR–BLG bioconjugates (bottom row, (**B**)).

The AuNRs had an average diameter of 13 ± 1 nm and a length distribution of 42 ± 2 nm (Figure S1). The AuNS–APO and AuNS–BLG bioconjugates were prepared using the same procedure as for the gold nanospheres (see Materials and Methods). Figure 2 shows the bright-field and the HAADF-STEM images, as well as the corresponding EDX elemental mapping of gold for the AuNR–APO and AuNR–BLG bioconjugates. The EDX analysis showing co-localisation of gold onto the fibrils confirmed the presence of the AuNRs associated with the amyloid fibrils. TEM imaging therefore provided evidence that the protein fibrils were successfully functionalised with AuNRs and that the medium was clean, i.e., there were no free AuNRs.

As mentioned previously, localized surface plasmon resonance (LSPR) is very sensitive to NP anisotropy. Rod-shaped AuNPs or nanorods are characterised by two LSPR bands: a transverse oscillation with a visible resonance corresponding to that of a sphere, and a longitudinal oscillation exhibiting a near infrared (NIR) resonance shifted to longer wavelengths and stronger intensities as the aspect ratio increases [8]. The UV-visible absorption spectra recorded for the AuNR–APO and AuNR–BLG fibrils both displayed two absorption bands centred at 530 nm and 750 nm (Figure 2). We can infer from the TEM and UV-vis measurements that the AuNRs are randomly distributed along the fibrils and that the proteins prevent nanoparticle aggregation and, therefore, further precipitation (Figure 2) for at least 1 month.

### 3.2. APO and BLG Amyloid Fibril-Biotemplated MNPs

**Figure 3:**
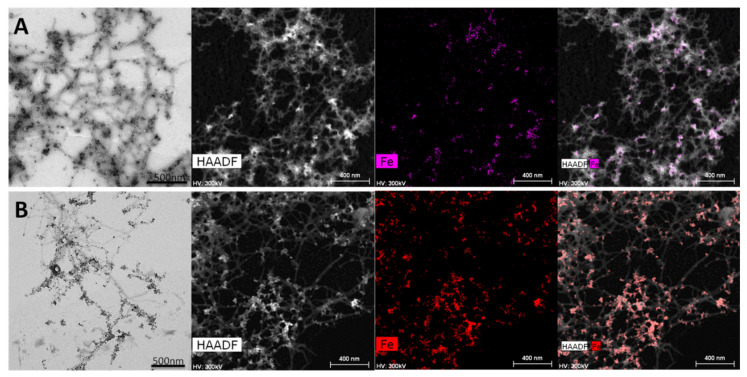
From left to right: TEM image, HAADF-STEM image, the corresponding elemental mapping of iron and the superposition of both images for MNP–APO (top row, (**A**)) and MNPs–BLG bioconjugates (bottom row, (**B**)).

Figure 3 shows the characterisation of the MNP–APO and MNP–BLG bioconjugates by TEM, and HAADF-STEM and the corresponding EDX elemental mapping of iron. The EDX mapping confirmed that the MNPs were effectively linked to the fibrils. The MNP–fibril assemblies were formed by incubating maghemite NPs (γ-Fe_2_O_3_, 8 nm average diameter, σ = 0.3, Figure S2) with the APO and BLG protein fibrils synthesised previously. TEM images showed that the MNPs were successfully incorporated into the fibril template and that there were no free particles in the medium. The presence of the fibril templates prevented the irreversible aggregation of the MNPs and their subsequent precipitation for at least after 1 month, as checked by TEM.

**Figure 4:**
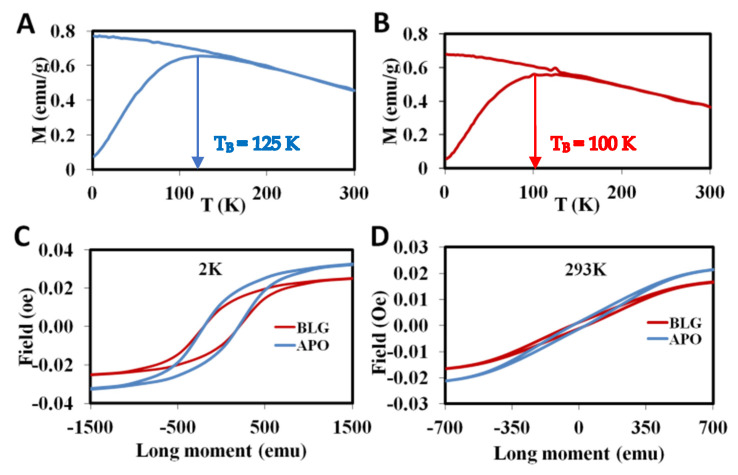
Zero-field cooled/field cooled curves recorded in a 50 Oe field (H) for (**A**) MNP–APO and (**B**) MNP–BLG bioconjugates. Hysteresis loops for MNP–APO and MNP–BLG samples at (**C**) 2 K and (**D**) 293 K.

Figure 4 shows the magnetic properties of MNP–APO and MNP–BLG bioconjugates. Zero-field cooled/field cooled (ZFC-FC) magnetisation curves were recorded as a function of temperature (2–300 K) in a field of H = 50 Oe (Figure 4A,B). As a common feature their shapes evidence a single maximum on the ZFC curve at a temperature T_B_ (blocking temperature), and increasing susceptibility when lowering the temperature on the FC curves. Such behaviour is characteristic of superparamagnetism. In each case when lowering the temperature, the FC curve follows the ZFC one and deviates at a temperature very close to T_B_. The T_B_ obtained from the maxima for the ZFC curves were 125 and 100 K for MNPs–APO and MNPs–BLG amyloid fibrils, respectively. Above T_B_ the system behaves as a superparamagnet and the ZFC and FC curves superimpose perfectly, so we can rule out the presence of any significant amount of aggregation. The T_B_ values were higher than those for similarly sized isolated γ-Fe_2_O_3_ NPs (90 K) [24,46]. This increase in T_B_ is due to the dipole-dipole interactions between NPs.

Figure 4C,D and Figure S3 show the hysteresis loops recorded at 2 K and 293 K for the MNP–APO and MNP–BLG bioconjugates. The coercive field (H_C_) and reduced remanence ratio (M_R_/M_S_) parameters indirectly reflect the system anisotropy. H_C_ was 250 Oe for both the MNP–APO and MNP–BLG fibril samples, compared to the pristine NPs which had a H_C_ of 175 Oe [24,47]. M_S_ values were 69 emu/gFe and 58 emu/gFe for MNPs–APO and MNPs–BLG fibrils, respectively. These values are comparable with M_S_ values for anisotropic iron oxide nanoparticles which showed high T2 values [47]. The r2 value is proportional to the square of two key parameters in highly magnetized nanomaterials: Ms value and effective radius of magnetic core. The reduced remanence values for randomly oriented γ-Fe_2_O_3_ NPs ranged between 0.2–0.3 [48]. The M_R_/M_S_ values were 0.43 and 0.4 for the MNP–APO and MNP–BLG fibrils, respectively. The increase in the M_R_/M_S_ and H_C_ values is a measure of the strength of the magnetic anisotropy and clearly indicates that γ-Fe_2_O_3_ NPs are characterised by uniaxial anisotropy. Again, this increase in the magnetic anisotropy of the MNP–fibril bioconjugates is probably due to the long-range dipole-dipole coupling and the MNPs alignment along amyloid fibrils. The magnetisation curves recorded at room temperature (293 K) showed negligible coercivity values for both bioconjugates, confirming their superparamagnetic behaviour. Higher M_R_/M_S_ and H_C_ values compared to pristine maghemite NP are a measure of the system’s magnetic strength. These collective results provide direct evidence that MNPs–protein fibrils could show potential to increase the r2 value due to their anisotropic nanostructure.

### 3.3. Metal-Binding APO Fibrils as Nanoreactors for the Preparation of Metal NPs–Protein Bioconjugates

In our second approach, we exploited the solvent-exposed metal-binding residues in APO amyloid fibrils as nanoreactors for the in situ synthesis of AuNP, AgNP and PdNP bioconjugates [49,50,51]. This approach is based on the high-affinity binding of some metal ions with the amyloid fibril functional groups and on the capacity of these bond metal ions to react with an appropriate reducing agent in order to nucleate the metal NPs in situ (Scheme 1B). This method could, therefore, produce a wide range of zero-valent nanoparticle–protein hybrid materials. The APO amyloid fibril acts as a scaffold and a stabilizer for NPs, thus preventing precipitation. Au nanoseeds were formed in situ on the protein fibrils with a high degree of order and alignment.

**Figure 5:**
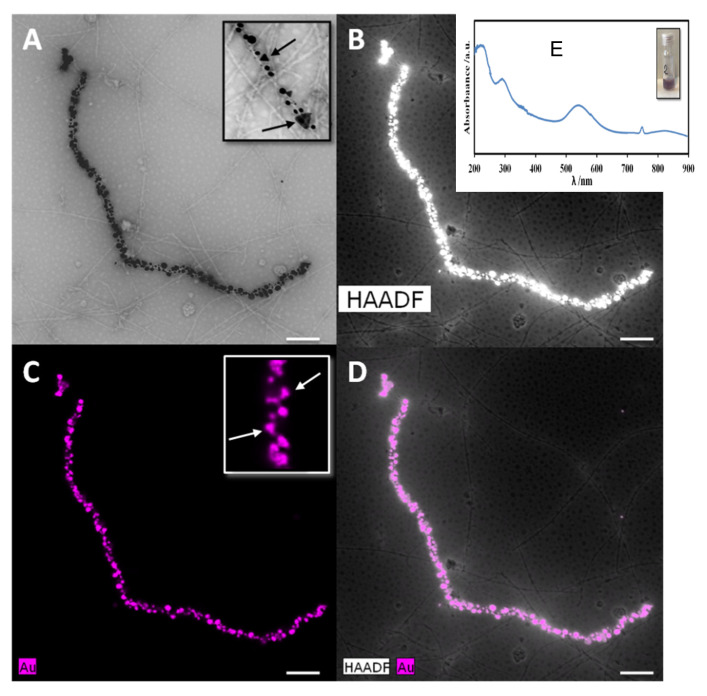
TEM (**A**), HAADF-STEM image (**B**), the corresponding elemental mapping of gold (**C**) and the superposition of both images for AuNP–APO fibrils (**D**). The presence of prisms is indicated with arrows in the insets. UV-vis spectrum of the AuNP–APO fibrils (**E**).

APO amyloid fibrils in the presence of gold salt were exposed to traditional metal reducing agents such as NaBH_4_ (Figure S4). Figure 5 shows TEM and HAADF- STEM micrographs of APO fibrils after incubation with HAuCl_4_ and subsequent reduction, and the corresponding EDX elemental mapping of gold. The AuNPs formed were simultaneously assembled on both sides of the fibrils (average diameter: 5 ± 2 nm). This produced AuNPs that were continuously aligned along the fibrils. The specific assembly of the AuNPs was fairly homogeneous on the APO-based amyloid fibrils, suggesting a uniform growth process from initial seeding to final formation. AuNPs–APO fibrils were stable in solution for at least after 1 month, as checked by TEM.

We confirmed the presence of near-spherical gold NPs bounded to the fibrils by UV-vis (Figure 5E). AuNP–APO fibrils displayed an absorption band attributed to the collective dipole oscillation at 530 nm. Interestingly, a broad expanded second band was observed at around 850 nm. The AuNPs interaction along the fibril arrangement cause a red shift of the longitudinal SPR band [52]. This new band corresponds to the AuNPs aggregation along the fibril as well as the presence of gold prisms as observed by TEM (Figure 5A,C, inset). Therefore, the SPR band can be tuned into the NIR region, which is where bioimaging techniques operate [8,53], thanks to the AuNPs assuming fibril-like structures.

To validate the easy, great potential and versatility of this straightforward second approach, we used similar methods to prepare AgNP– and PdNP–APO bioconjugates, by incubating silver or palladium salts with the APO protein fibrils followed by in situ reduction, akin to previous work on BLG protein [40]. Due to the regular distribution of the chemical groups along the APO protein surface, these self-assembled biotemplates can functionalise with a broad range of NPs, molecules or metal ions. These samples were characterised by TEM, EDX and UV-vis spectroscopy (Figures S5 and S6). TEM images revealed the presence of very small PdNPs (5 ± 1 nm), with the NPs arranged alternately along the fibrils. However, in the case of silver, the NPs presented a broad size distribution, ranging from 20 to 70 nm. In addition, the medium was once again clean for both samples. EDX confirmed the presence of palladium and silver (Figure S5).

## 4. Conclusions

In this study, we have shown how APO amyloid fibrils can serve as bio-caffolds for the preparation of metal NP–protein bioconjugates, and compared them to a BLG amyloid protein model. In the first approach, AuNP–protein bioconjugates were prepared by direct coupling of AuNPs with different morphologies to APO and BLG amyloid proteins. Protein fibril templates were previously prepared through a temperature-induced self-assembly process. The plasmonic properties exhibited by the AuNP–protein fibrils mean they are potential candidates for use as optical imaging enhancers based on their LSPR absorption bands. Moreover, the strong band in the NIR region for the AuNR–protein bioconjugate could find applications in tumour hyperthermia treatment.

By using amyloids self-assembled from APO and BLG as biotemplates we prepared the MNP–APO and MNP–BLG–protein magnetic bioconjugates starting from γ-Fe_2_O_3_ NPs. During this self-assembly process, the protein binds the MNPs together, preventing precipitation and massive aggregation. SQUID measurements confirmed the bioconjugates superparamagnetism, required for in vivo applications. This improvement in magnetic anisotropy is probably due to 1D fibril organisation and dipole-dipole interactions. Assembling NPs into fibrillar structures to enhance magnetic properties is a genuine strategy towards increasing the MRI contrast effect.

In our second approach, we synthesized AuNP–protein bioconjugates by in situ gold chemical reduction of metal ions in the presence of the APO amyloid fibrils. The specific assembly and the high degree of alignment of fairly homogeneous AuNPs onto APO–protein fibrils suggest a uniform growth process. To illustrate the general nature of the approach, we also prepared AgNP– and PdNPs–protein bioconjugates. Thanks to the regular distribution of the solvent-exposed metal-binding residues on the surface of the APO amyloid fibrils, a broad range of NPs, molecules or metal ions can be functionalised.

To summarise, the controlled arrangements of NPs on biological templates can form hybrid inorganic-organic materials that can be adapted to practical applications, particularly in the area of bioimaging. In these systems, the physical properties of the material can be tuned by modifying the nanoparticle composition, shape and size, while mechanical, elastic and light-weighted properties can be adjusted by selecting different biological templates. As a proof of concept, we have shown that APO protein amyloid fibrils can be used as scaffolds to prepare different metal biomaterials, where the inorganic components and their mutual arrangement within the assembled structure define their final properties.

## Supplementary Materials

The following are available online at https://www.mdpi.com/2079-4991/11/1/146/s1, Figure S1: TEM images of gold spherical nanoparticles (left) and gold nanorods (right); Figure S2: TEM image of maghemite nanoparticles; Figure S3: Magnetic hysteresis loops of MNP–APO and MNP–BLG samples at 2 and 293 K; Figure S4: AgNPs and PdNP–APO before (a) and after (b) the corresponding salt reduction; Figure S5: TEM images and EDX spectra of (a) AgNP–APO and (b) PdNP–APO fibrils, Figure S6: UV-vis spectrum of AgNP–APO fibrils. The insert shows the corresponding brownish sample of AgNP–APO fibrils.
