# Supplementary material for: Apoferritin Amyloid-Fibril Directed the In Situ Assembly and/or Synthesis of Optical and Magnetic Nanoparticles
Source: Nanomaterials (Basel). 2021 Jan 8;11(1):146. doi: 10.3390/nano11010146 (PMC7826742; doi:10.3390/nano11010146)
Supplement: Supplementary file 1 [file nanomaterials-11-00146-s001.pdf]

Supporting Information

Apoferritin Amyloid-Fibril Directed the In Situ Assembly and/or Synthesis of Optical and Magnetic Nanoparticles

Rocío Jurado and Natividad Gálvez *

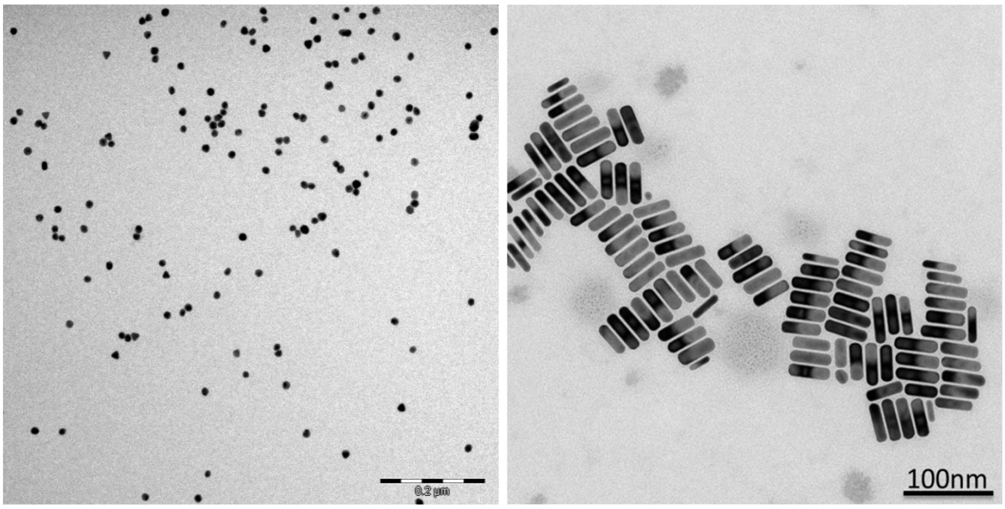

Figure S1. Gold spherical nanoparticles (**left**) and gold nanorods (**right**) TEM images.

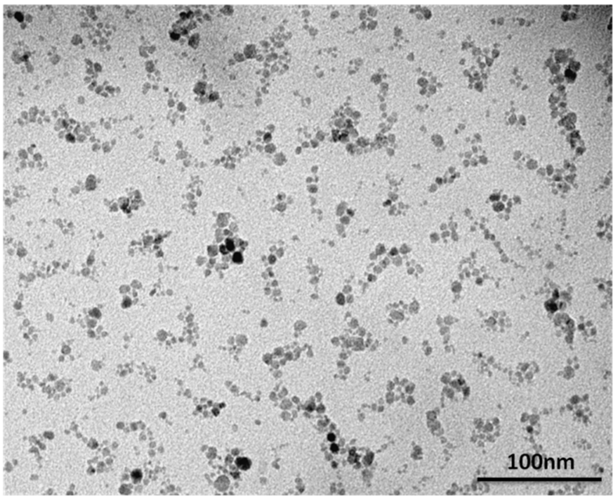

Figure S2. Maghemite nanoparticles TEM image.

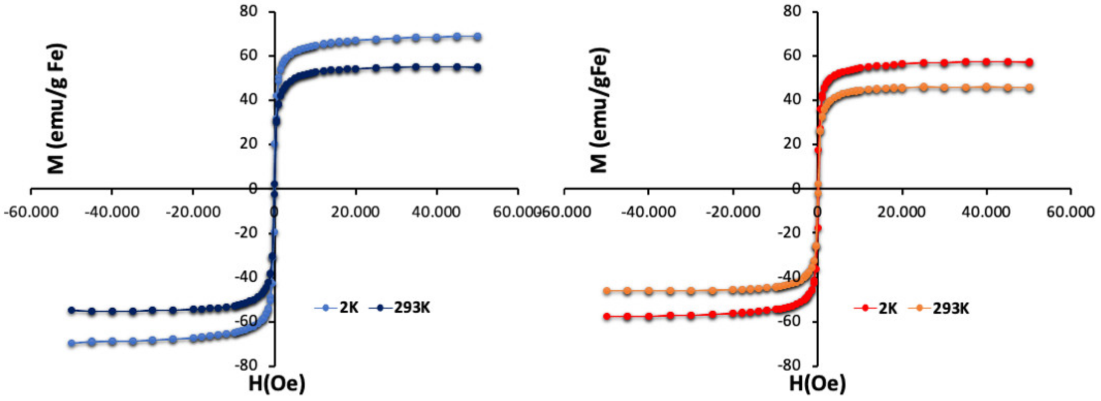

Figure S3. Magnetic hysteresis loops of (left in blue) MNPs-APO and (right in red) MNPs-BLG samples at 2 and 293K.

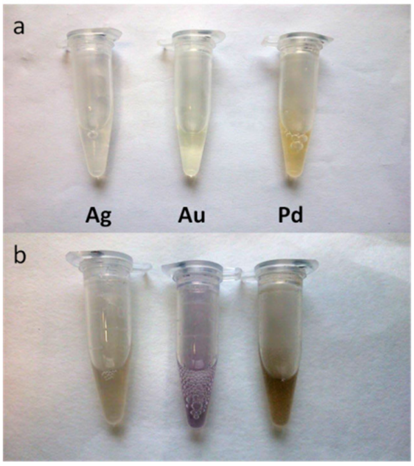

Figure S4. AgNPs- and PdNPs-APO before (**a**) and after (**b**) the corresponding salt reduction.

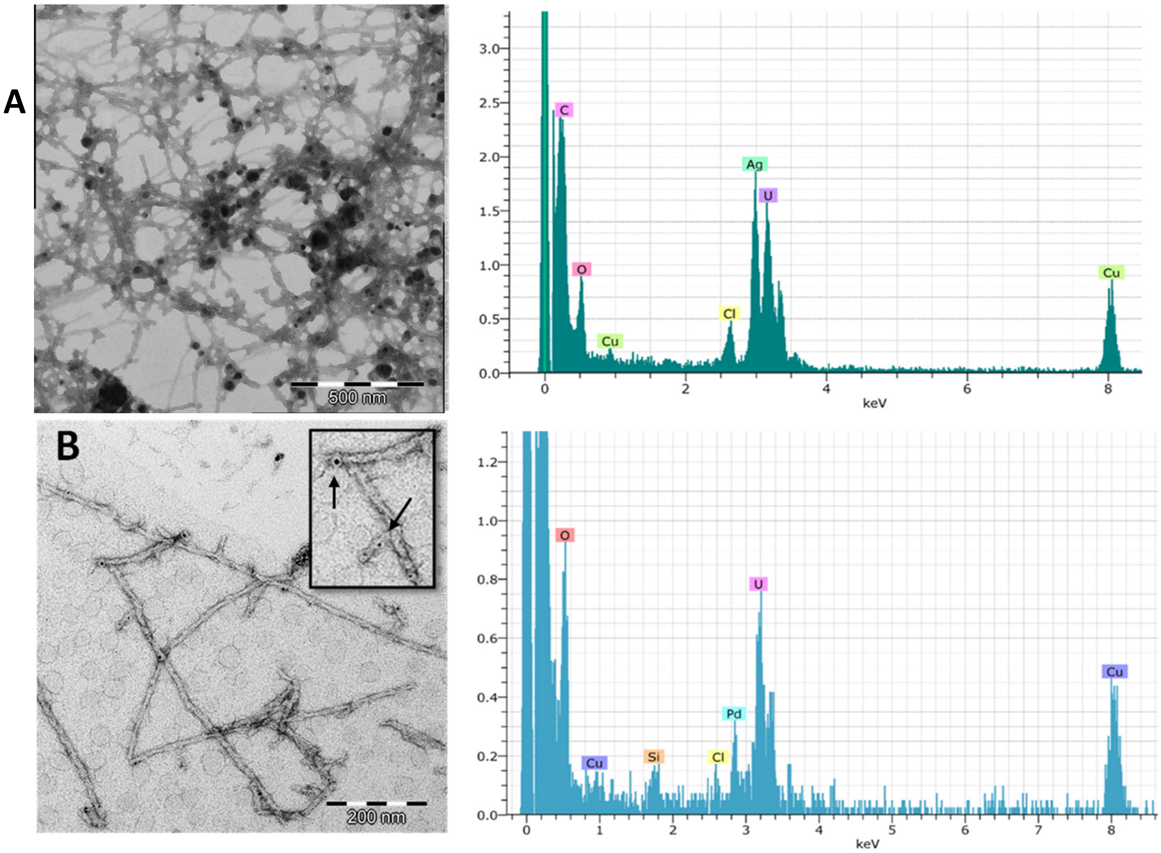

Figure S5. TEM images and EDS spectra of (**a**) AgNPs-APO and (**b**) PdNPs-APO fibrils.

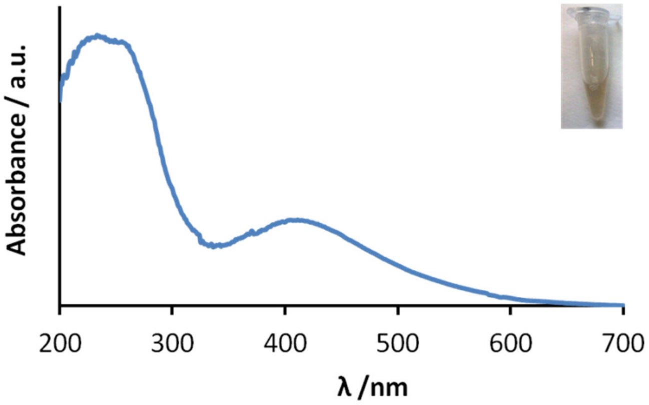

Figure S6. UV-vis spectrum of AgNPs-APO fibrils. The insert shows the corresponding brownish sample of AgNPs-APO fibrils.
